# Effect of Pressure Loading on Heel Skin Temperature and Moisture in Healthy Volunteers: Preliminary Results

**DOI:** 10.3390/biology13100782

**Published:** 2024-09-29

**Authors:** Érica de Carvalho, Leticia Costa Marostica, Gabriela Fagundes Trento, Julia Scalco Marcolina, Fernanda Ceolin Teló, Rhea Silvia de Avila Soares, Lidiana Batista Teixeira Dutra Silveira, Suzinara Beatriz Soares de Lima, Paulo Jorge Pereira Alves, Thaís Dresch Eberhardt

**Affiliations:** 1 São Vicente de Paulo Hospital, Passo Fundo 99010-080, RS, Brazil; ericadecarvalho34@gmail.com; 2 São João Charitable Hospital, Sananduva 99840-000, RS, Brazil; marostica97leticia@yahoo.com; 3 Institute of Heatlh, Universidade de Passo Fundo, Passo Fundo 99052-900, RS, Brazil; 92630@upf.br (G.F.T.); 183792@upf.br (J.S.M.); ferceolintelo@gmail.com (F.C.T.); 4 Polytechnic College, Universidade Federal de Santa Maria, Santa Maria 97105-900, RS, Brazil; rheasilviasoares@yahoo.com.br; 5 Postgraduate Nursing Program, Universidade Federal de Santa Maria, Santa Maria 97105-900, RS, Brazil; lidianadutrasilveira@gmail.com; 6 Nursing Department, Postgraduate Nursing Program, Universidade Federal de Santa Maria, Santa Maria 97105-900, RS, Brazil; suzibslima@yahoo.com.br; 7 Centro de Investigação Interdisciplinar em Saúde, Faculty of Health Sciences and Nursing, Universidade Católica Portuguesa, 4169-005 Porto, Portugal; pjalves@ucp.pt; 8 Postgraduate Program in Bioexperimentation (PPGBioexp), Institute of Heatlh, Universidade de Passo Fundo, Passo Fundo 99052-900, RS, Brazil

**Keywords:** pressure ulcer, adult, heel, skin, skin temperature, epidermis

## Abstract

**Simple Summary:**

The skin microclimate can be defined as the relationship between temperature and moisture of the skin surface, being considered a risk factor for the development of pressure injuries. The objective was to evaluate the effect of pressure loading on skin temperature and moisture in the heel region of healthy adults. The study was performed at the University of Passo Fundo, Brazil, in October 2022. Ten individuals were evaluated. The pressure loading leads to a decrease in temperature and changes the skin moisture of the heels of healthy individuals.

**Abstract:**

Recent studies emphasize the significance of skin microclimate in the prevention of pressure injuries (PI). The objective was to evaluate the effect of pressure loading on skin temperature and moisture in the heels of healthy adults. This is a before-and-after study performed at Brazil, in October 2022. Skin temperature (°C) was measured by an infrared digital thermometer, and skin moisture (%) using electrical bioimpedance. Ten individuals/twenty heels were evaluated. The average temperature of the right and left heel was the same at baseline (27.2 °C). It was recognized that after 30 min of pressure loading on the heels, there was a decrease in temperature, and after 15 min of pressure offloading, the temperature decreased again. It was found that at t0, the moisture of the right heel (12.6%) was lower than the left heel (15.6%). The median moisture in the right heel increased from t0 to t1 and decreased in t2, while in the left heel, there was a small variation of the median from t0 to t1, as well as to t2. The pressure loading leads to a decrease in temperature and changes the skin moisture of the heels of healthy individuals.

## 1. Introduction

The microclimate of the skin can be defined as the relationship between temperature and moisture of the skin surface [1] and can be considered a risk factor for the development of pressure injury (PI), as it interferes with the soft tissue tolerance to pressure and shear [2].

Despite the scarcity of scientific studies that relate the microclimate and its effects on the risk of developing PI, it is known that the temperature and high moisture of the skin can affect metabolic demands, as well as the mechanical behavior of the tissue. From this perspective, it is understood that contact with clothing, bed position, chair, dressings or medical devices alter the microclimate of the skin [2].

Pressure injuries in heels remain a significant concern in healthcare settings. In one National Health System (NHS) trust, heel pressure ulcers accounted for 71% of avoidable category 3 and above PIs, prompting a quality improvement project that reduced incidence over three years [3]. In hospital emergency services, heel pressure ulcers are among the most problematic anatomical locations, with prevalence ranging from 5.2% to 12.3% and incidence from 4.5% to 78.4% [4]. Furthermore, it is highlighted that this is due to the anatomical characteristics of the heels, because they have a curved and accentuated shape affecting the soft tissues [5].

Recent research has highlighted the importance of skin microclimate in PI development and prevention. A study investigated how dressings affect microclimate conditions on weight-bearing skin, finding that the thermal properties of dressings play a crucial role in mitigating PI risk [6].

Infrared thermography has been used to measure skin temperatures, revealing that lying down for one hour can cause significant heat-trapping between the body and the support surface [7]. Another study also showed an increase in skin temperature in the sacral and trochanteric regions with the application of pressure [8]. In addition, there is evidence to indicate that an increase in skin temperature leads to changes in skin structure and function [9].

Skin moisture is also a factor that influences the development of PI, mainly related to incontinence [10]. Still, the application of pressure on the heels increases skin moisture [11]. It is noteworthy that a systematic review revealed mixed results, with only 33.3% of studies reporting a statistically significant association between skin hydration and PI development [12].

However, the ideal microclimate values on the skin surface and how to achieve it remain unknown in the literature [2]. Given the above, this study aims to evaluate the effect of pressure loading on temperature and skin moisture in the heel region of healthy individuals.

## 2. Materials and Methods

This is a before-and-after study in which the variables were measured before and after an intervention. This study was part of the matrix project entitled “Microclimate of the skin of different body areas in healthy adults: clinical parameter for prevention of pressure injuries”.

The research was conducted at the Center for Realistic Simulation (CRS) of the University of Passo Fundo, Brazil. The CRS is a space that contains technologies for teaching in the health area, allowing students and professionals to develop clinical skills and learn to perform health procedures, with the use of realistic simulation and high-fidelity equipment.

The participants were healthy adults aged 18 to 59 with no diagnosed medical conditions. The sample size was calculated using G Power 3.1 [13], with a paired *t*-test [14], assuming an effect size of 0.5, 90% power and a significance level of 99% (α < 0.01). This resulted in a sample size of 55, with an additional 30% (17 individuals) added to account for potential dropouts.

For the recruitment of participants, the dissemination among the academic community by e-mail was carried out by the responsible sectors of the university. In addition, posters were printed, which were placed in areas of movement of people on the campus, as well as disseminated through social networks. The information contained a WhatsApp number to schedule the data collection and provide explanations on the research.

The collection team was composed of five nursing students. Before the beginning of the data collection, the theoretical and practical training of the collectors was carried out in the CRS, lasting two hours. Moreover, a pilot test was conducted. As no changes were necessary in the data collection instrument, the tested individual was included in the research.

Data collection took place in October 2022, with successive entries of individuals. Samples were collected during the day, between 8:00 a.m. and 6:00 p.m., due to differences in body temperature that may occur during the day as a function of circadian rhythm.

At first, hand hygiene was performed, following the recommendations of the National Health Surveillance Agency. Soon after, the disinfection of the stretcher using liquid alcohol 70% and disposable multipurpose cloth was carried out. Again, hand hygiene was performed, and a paper sheet was placed on the stretcher.

Subsequently, the research participant was instructed to lie on the stretcher in a right lateral position (baseline—t0). The collection of variables (body temperature, skin temperature and moisture, temperature, and humidity of the environment) began with the measurement of the same variables before the intervention (pressure). Then, the individual was instructed to stay in the supine position for 30 min, with pressure on the heels (t1), and a new measurement of the variables was performed after that time. Then, the participant remained for 15 min again in the right lateral position, with pressure on the heels (t2), followed by measurement again. During the entire collection period, the heels were exposed, with participants not wearing socks or shoes.

It should be noted that after data collection, the equipment used was disinfected with a tissue moistened with 70% alcohol. The paper sheet was discarded, and the stretcher was disinfected and hand hygiene performed.

The outcome of this research was the temperature (°C) and skin moisture (%) of the heels. This was measured using an infrared digital thermometer (62 MAX, Fluke Corporation, Everett, DC, USA). The device checks the temperature in degrees Celsius or Fahrenheit with a single-point laser. The distance between the skin and the thermometer was seven centimeters, in accordance with a similar survey conducted in Indonesia [15]. The choice for the brand of the thermograph was made in accordance with another study that used an infrared device of the same brand [15].

The variable skin moisture was measured using electrical Bioimpedance (Skin Analyser SKN1501, Skin Up Beauty Devices, Shenzhen, China). It should be noted that this device is considered accurate to measure this variable [16]. Both these devices were calibrated before data collection began.

Regarding the independent variables of the study, patient identification was removed from the database to preserve the identity of the research participant, being replaced by a code as the input in the study (A1, A2, A3…). Age (in full years), gender, and self-declared race were collected.

The variables of temperature (°C) and humidity (%) of the environment were also collected using a thermohygrometer (model 7663.02.0.00, Cotronic Technology Ltd., Shenzhen, China) every time skin temperature was measured. The device was calibrated previously. For the measurement of body temperature (axillary), a clinical digital thermometer was used.

**Figure 1:**
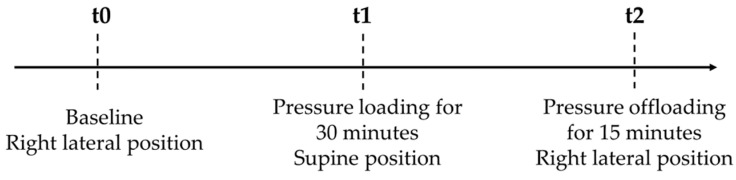
Data collection points. Passo Fundo—RS, Brazil, 2022.

Figure 1 shows the moments of data collection of skin temperature, body temperature, temperature, and humidity of the environment. All other variables were measured at baseline.

The collected data were recorded in a double-blind and independent manner in a Microsoft Office Excel^®^ 365 Spreadsheet and analyzed in the same way. A simple descriptive statistical analysis was performed. The quantitative variables were described by measures of central tendency (mean or median) and dispersion (standard deviation or interquartile range), according to the coefficient of variation.

The study was approved by the Ethics Committee of the Passo Fundo University with the Certificate of Presentation for Ethical Appreciation (CAAE) 59270822.5.0000.5342 and report number 5.582.712.

## 3. Results

For this study, 10 individuals (20 heels) were evaluated. The sociodemographic characteristics of the study participants are described in Table 1. All participants were female (n = 10; 100.0%) and the majority were White (n = 7; 70.0%) with a mean age of 24.2 years.

Table 2 shows the data of heel temperature at collection times t0, t1, and t2 and the differences between each collection point. The average temperature of the right and left heel was the same at baseline (27.2 °C). It was identified that after 30 min of pressure loading on the heels, there was a decrease in temperature (mean = 1.0 °C). After 15 min of pressure offloading, the temperature decreased again (mean = 1.1 °C)

Table 3 presents data on skin moisture of the right and left heels at the time of data collection. It was found that at t0, the moisture of the right heel (12.6%) was lower than that of the left heel (15.6%). Moreover, there was an increase in moisture in the right heel (5.0%) and a decrease in the left heel (−4.9%) after the loading of pressure for 30 min. After 15 min of pressure offloading, there was a decrease in moisture in the right heel (−6.5%) and a small change in the left heel (0.6%).

**Figure 2:**
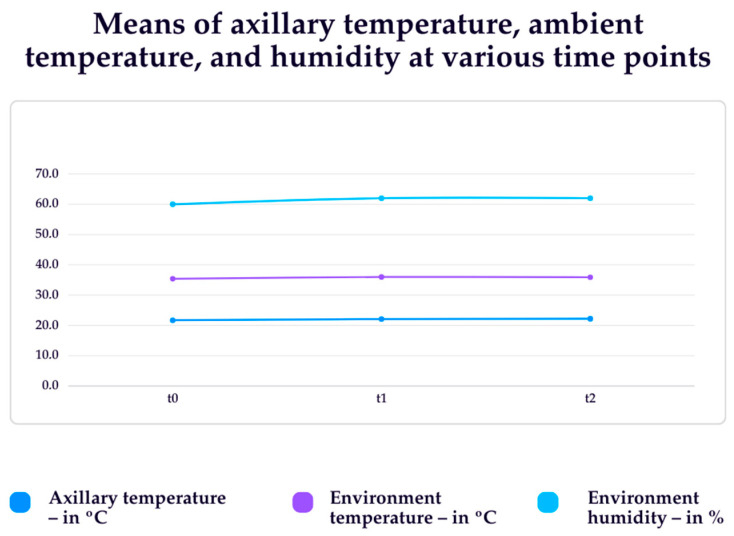
Means of axillary temperature, ambient temperature, and humidity at various time points (n = 10). Passo Fundo—RS, Brazil, 2022.

Figure 2 presents data related to axillary temperature, environment temperature, and humidity in the collection times t0, t1, and t2. It was observed that the axillary temperature ranged from 34.1 to 36.8 °C; the ambient temperature ranged from 19.6 to 23.5 °C; and the ambient humidity ranged from 55.0 to 70.0%.

## 4. Discussion

The participants in this study were all female, mostly white, with a mean age of 24.2, and were all healthy young adults. It should be noted that the researchers did not set a requirement for the inclusion of only women; however, this was the profile of the individuals who volunteered to participate in the study.

It is noteworthy that symmetry was identified between the body sides at t0, and the average temperature in both heels was 27.2 °C. Another study identified the average heel temperature as 28.2 °C in hospitalized individuals without risk of developing PI [17]. In addition, other studies also found symmetry in body temperature between the right and left sides [18].

However, in t1 and t2, we identified different average temperatures between the body sides. Similar data were found in another study that evaluated the effect of pressure loading in healthy individuals and identified the existence of changes in heel skin temperature between the two legs over time [19].

The average temperature in heels after 30 min of pressure loading (t1) was 26.2 °C, with an average decrease of 1.0 °C. Moreover, it was identified that pressure offloading for 15 min also led to a decrease in skin temperature, on average 1.1 °C.

Some scientific evidence reinforces this finding, indicating that a decrease in skin temperature can lead to the appearance of lesions. One study identified that the temperature of colder skin increases the chance of necrosis, and individuals who developed PI had lower average skin temperature than individuals who did not develop this type of lesion [20]. However, other studies have brought controversial results to this discussion by demonstrating that pressure loading causes increased skin temperature [21]. Consequently, increased skin temperature can increase the risk of developing PI [1].

As for the skin moisture of the heels, it was identified that there is no body symmetry, as the right heel (12.6%) had a lower basal median moisture than the left (15.6%). The effect of pressure loading for 30 min and pressure offloading for 15 min was also different between the body sides. In the right heel, after pressure loading, there was a median increase of 5.0% of moisture and, after pressure offloading, a median decrease of 6.5%.

This result is aligned with the study that shows that tissue damage generates increased moisture in the skin, and that a high level of moisture creates changes in impedance, potentiating pressure lesions at the site [22]. In addition, this increase in the median moisture of the right heel may be associated with the selection of only female individuals.

Therefore, it was observed that in females, skin–material friction is higher, as these individuals are more sensitive to changes in moisture. Similarly, women in one study were more likely to soften the skin strata and increase contact surface with materials [23]. In the left heel, after the pressure loading, there was a median decrease of 4.9% of moisture and, after pressure offloading, the variability was minimal (median of 0.6%). It should be noted that the heels are located in the region of the human organism that has a lower probability being oily, so it is less likely to increase moisture due to the less hydrophilic characteristics of the region [24]

Still, to understand this low variability of moisture after pressure offloading, the literature points out that advanced age is one of the characteristics for low values of skin moisture [25]. However, in this study, only young adults were selected. The selection of healthy individuals (absence of diseases) is also a characteristic that may possibly explain the low variability of moisture in the left heel.

Therefore, the literature points out that healthy tissue has a uniform shape and low moisture values [26]. In the United Kingdom, technologies that evaluate the subepidermal moisture of the skin to prevent pressure injuries are becoming increasingly widespread [26].

Concerning the average temperature of the environment, in this study, the variation ranged from 19.6 to 23.5 °C. A study that investigated the main sites of the human body for heating and cooling identified that with the increase in the ambient temperature from 25 to 31 °C, local skin temperatures also increased gradually [27].

It should be noted that skin temperature is an objective measure for assessing the risk of developing PI, so it should be evaluated as a parameter. Therefore, the results contribute to increasing our knowledge about the normal temperature parameters of the heel.

However, this study has some limitations, such as the absence of the measurement of the pressure loading, that is, the pressure may have been different due to the weight and shape of the individual’s body. Knowing the pressure load on the heels would allow for the analysis of whether different pressure loads can produce varying changes in heel skin temperature. Therefore, caution is needed when generalizing the findings of this study. In addition, the study presented only partial results due to the small number of participants, which did not allow for inferential statistical analysis.

Thus, new research is suggested to compare and evaluate the measure of pressure loading in healthy individuals and the influence of environmental temperature and humidity on skin temperature.

## 5. Conclusions

The average temperature found in the heel region of healthy individuals was 27.2 °C. Pressure loading for 30 min and the consequent offloading of the same for 15 min led to a decrease in skin temperature. Furthermore, it was identified that in t0, the moisture of the right heel (12.6%) was lower than that of the left heel (15.6%), that is, symmetry between the body sides was not identified. Moreover, there was an increase in moisture in the right heel and a decrease in the left heel after 30 min of pressure loading. After 15 min of pressure offloading, there was a decrease in the moisture of the right heel and a small change in the left heel. Therefore, from the partial data presented, it is understood that the pressure loading can interfere with the moisture values of the skin. In addition, pressure offloading showed a tendency to decrease skin moisture.

## Figures and Tables

**Table 1:** Distribution of sociodemographic variables of the research participants about the effect of pressure loading on the temperature of the heel skin in healthy individuals (n = 10). Passo Fundo—RS, Brazil, 2022.

Variables	n (%)
Sex	
Female	10 (100%)
Male	0 (0%)
Race	
White	7 (70.0%)
Brown	2 (20.0%)
Black	1 (10.0%)
Variable	Mean ± SD (Min–Max)
Age	24.2 ± 5.1 (20–33)

SD: standard deviation. Min: minimum. Max: maximum.

**Table 2:** Distribution of temperature in the heel region (n = 20). Passo Fundo—RS, Brazil, 2022.

Variables	Body Side		
	Right	Left	Mean Between Sides
	**Mean ± SD** **(95% CI)**	**Mean ± SD** **(95% CI)**	**Mean ± SD** **(95% CI)**
Heel temperature at t0 (baseline)—in °C	27.2 ± 2.6 (25.3–29.1)	27.2 ± 2.7 (25.3–29.2)	27.2 ± 2.6 (25.3–29.1)
Heel temperature in t1 (after 30 min of pressure loading)—in °C	26.4 ± 1.9 (25.0–27.7)	26.0 ± 2.0 (24.5–27.2)	26.2 ± 2.0 (24.8–27.4)
Heel temperature in t2 (after 15 min of pressure offloading)—in °C	25.4 ± 1.8 (24.1–26.7)	24.8 ± 1.7 (23.6–26.0)	25.1 ± 1.7 (23.9–26.3)
Difference between t1 and t0	−0.8 ± −0.7 (−1.7–0.0)	−1.2 ± −0.7 (−2.3–−0.4)	−1.0 ± 0.7 (−2.0–−0.2)
Difference between t2 and t1	−1.0 ± −0.1 (−1.3–−0.5)	−1.2 ± 0.3 (−1.4–−0.5)	−1.1 ± −0.2 (−1.5–−0.5)

SD: standard deviation. CI: confidence interval.

**Table 3:** Distribution of moisture in the heel region (n = 20). Passo Fundo—RS, Brazil, 2022.

Variables	Body Side			
	Left		Right	
	Median (IR)	Min–Max	Median (IR)	Min–Max
Heel moisture at t0 (baseline)—in %	12.6 (26.3)	10.1–54.5	15.6 (26.3)	10.3–61.7
Heel moisture at t1 (after 30 min of pressure loading)—in %	17.6 (17.2)	10.1–53.2	10.3 (0.0)	10.3–10.3
Heel moisture in t2 (after 15 min of pressure offloading)—in %	11.1 (3.3)	10.2–39.1	10.7 (13.2)	10.2–30.7
Difference between t1 and t0	5.0 (−9.1)	−25.8–14.4	−4.9(−13.1)	−49.7–7.2
Difference between t2 and t1	−6.5 (−14.0)	−41.1–15.7	0.6 (6.4)	−1.7–17.4

IR: interquartile range. Min: minimum. Max: maximum.

## Data Availability

Data are contained within the article.
